# Caregivers’ lived experiences of childhood probable pneumonia through a gendered lens in western Kenya

**DOI:** 10.4102/phcfm.v17i1.4758

**Published:** 2025-07-08

**Authors:** Sarah Hawi Ngere, Charles Olang’o, Kennedy Ochola, Patience Oduor, Caleb K. Sagam, Benard Ochieng, Dickens Omondi, Norbert Peshu, Erick Nyambedha

**Affiliations:** 1 Kenya Medical Research Institute-Centre for Global Health Research, Kisumu, Kenya; 2 Department of Sociology and Anthropology, Faculty of Arts and Social Sciences, Maseno University, Kisumu, Kenya; 3 KEMRI-Wellcome Trust Research Programme, Kilifi, Kenya; 4 Africa Bioethics Network, Nairobi, Kenya; 5 School of Health Sciences, Department of Biomedical Sciences and Nursing, Jaramogi Oginga Odinga University of Science and Technology, Siaya, Kenya

**Keywords:** gender, intersectionality, lived experiences, children, hermeneutic phenomenology, Kenya

## Abstract

**Background:**

Gender intersects with multiple forms of socio-cultural, economic and health system factors to influence the overall care-seeking experiences of caregivers.

**Aim:**

This study aimed to understand the multiple gendered intersecting factors that shape women caregivers care-seeking experiences for children with probable pneumonia.

**Setting:**

The study was conducted in Karemo, Siaya County in western Kenya.

**Methods:**

In-depth interviews (IDI), participant observation and informal interviews were utilised. The IDI was conducted among purposively selected 12 caregivers out of which 11 were enrolled in participant observation. Data were managed and analysed using Dedoose and hermeneutic phenomenology, respectively.

**Results:**

Women juggle household chores, caregiving and income-generating activities, which sometimes led them to decline child’s hospitalisation because of competing household responsibilities. At the hospital, women experienced long waiting times, poor communication, unfavourable conditions, unfriendly staff and lack of drugs. Some women reported challenges in accessing money from their husbands for their child’s healthcare. They were often required to make difficult choices, such as prioritising work because of financial constraints, prioritising other children because of lack of external support, or opting for over-the-counter medication because of convenience, drug shortages or long waiting times at the hospital.

**Conclusion:**

Lived experiences are shaped by women’s daily realities and constraints. To improve women’s caregiving experiences, a holistic approach that considers the multiple dimensions of caregivers’ lives and gendered dynamics is recommended.

**Contribution:**

This study’s findings emphasise the necessity of a holistic approach when developing intervention geared towards improving healthcare-seeking behaviour by considering the subtler factors beyond structural, social and economic influences.

## Introduction

Gender encompasses socially constructed norms, behaviours and roles associated with being identified as a woman, man, boy or girl, and includes the dynamics that exist between them. These norms are not static and can vary significantly across different societies, evolving over time.^1^ Gender hierarchies contribute to inequalities that intersect with other social and economic disparities, a phenomenon well-documented in global health contexts.^1,2^ Gender perspective therefore looks at the impact of gender on people’s opportunities, social roles and interactions.^3^ However, it is the intersection of gender and the various social, cultural and economic factors that shape individual experiences in the context of care-seeking. Researchers argue that using intersectionality approaches enables the understanding of the complexity of the interactions between social stratifiers.^4,5,6^

Past research has shown that the intersection between economic factors and gender roles acted as barriers to care-seeking for children with pneumonia.^7,8^ Gender roles are constructed into behavioural expectations based on biological sex. Traditionally, men are expected to display masculine traits such as strength and power and less openly display emotion and affection.^9^ In most cases, women are expected to do domestic work which is considered feminine, such as caring for the children, household chores and working in the farm and other roles ascribed to women depending on the cultural context.^2^ Even when women work outside the home, traditional gender roles still apply in many contexts.^10,11^ For instance, male partners often make decisions concerning seeking treatment outside the home and provision of money to facilitate the process, despite women being the primary caregivers.^7,8,12^ Consequently, women may lack decision-making authority concerning their children’s healthcare needs.^13,14,15^ The impact of the intersection between gender roles and financial constraint is evident when women decline hospitalisation for sick children out of fear of lost wages.^16,17,18^

Socio-cultural factors and gender roles significantly influence care-seeking experiences, although these vary greatly depending on the context.^7,8^ In Bangladesh, gender-related influences discourage women from seeking healthcare from male doctors, highlighting the impact of cultural norms on healthcare decisions. Additionally, social expectations in Bangladesh mean that men are often unable to provide care for their children during hospital admissions, further reinforcing traditional gender roles where caregiving responsibilities within hospital wards predominantly fall on women.^19^ In Kebbi, Nigeria, societal restrictions on women’s mobility and interactions mean that fathers often become the primary care-seekers of healthcare services, while mothers remain the primary caregivers.^7^ In Nairobi, Kenya’s informal urban settlement, women typically hold decision-making power regarding their children’s healthcare, consulting fathers only when they face financial constraints in accessing medical care.^20^ Generally speaking, the interaction between gender dynamics and socio-cultural values is what shapes a gendered experience during care-seeking, these nuances are important to understand care-seeking experiences.

The World Health Organization (WHO) framework for improving the quality of paediatric care framework highlights the need to: (1) communicate with children and their families to enhance meaningful participation; (2) respect, protect and fulfil rights of every child at all times during care, without discrimination; and (3) provide all children and their families with educational, emotional and psychosocial support that is sensitive to their needs and strengthens their capability.^21^ However, in hospitals, women, due to their caregiving roles, are more likely to be the ones caring for children during hospitalisation. They often encounter long queues, lack of an effective triage system and unprofessional behaviour from health workers.^22,23,24^ Moreover, these women frequently experience negative power dynamics that hinder effective communication between caregivers and healthcare providers.^25,26^ During hospitalisation caregivers are expected to be involved in the sick child’s care; however, the discussion about their sick child with the medical practitioners is minimal. This therefore causes anxiety and inability of caregivers to cope in the difficult situation.^27^ Additionally, caregivers are often sent home without a proper diagnosis or treatment, and parents are not adequately informed about the danger signs to watch for in their children.^28^

The influence of gender and its intersection with other social stratifiers for prevention and care-seeking has been investigated in other studies.^4,5,19,29^ However, research to identify gender and its intersecting factors related to caregiver’s care-seeking experiences for probable pneumonia is limited in Kenya. Given the intersectionality of gender norms, economic status, structural and socio-cultural factors, understanding the nuances of intersectionality reveals critical insights into the dynamics of care-seeking, healthcare access and social expectations. This approach highlights how various social positions influence individuals’ care-seeking experiences.

## Research methods and design

### Study design

The study adopted a qualitative design with an ethnographic approach, utilising the philosophical paradigm of Martin Heidegger’s hermeneutic phenomenology. This framework allowed for a deep exploration of the lived experiences of caregivers, aiming to uncover the meanings and interpretations ascribed to these experiences. By focusing on the caregivers’ perspectives and contexts, the study sought to provide a rich, nuanced understanding of the gendered nature of care-seeking experiences. The data presented in this paper came from a larger ethnographic study seeking to understand caregiver’s perspectives and care-seeking pathways for probable pneumonia for children under 5 years. A mix of qualitative data was used, including in-depth interviews, participant observation and informal interviews.

### Study area

The study took place in Opanga village, Karemo division, within the Health Demographic Surveillance System (HDSS) area in Siaya County. As of 2022, unpublished data report that Karemo division had 11 464 children under five, with 1674 (14.6%) child deaths, 234 (14%) of which were because of pneumonia. The main health facility, Siaya County Referral Hospital (SCRH), has 200 inpatient beds, and there are 23 health facilities in Karemo Division. Ramba Pundo village, near Siaya township and close to SCRH and private hospitals, has an estimated population of 5122.

### Study participants

The study participants were caregivers, which included mothers, fathers or grandparents of children less than 5 years in Ramba Pundo village. The study team initially screened participants at their homes. The following questions were asked: (1) Did the child have a cough in the last 2 weeks; (2) Did the child have an illness with a cough, did he or she breathe faster than usual with short, rapid breaths or have difficulty in breathing; (3) Was the breathing fast or was it difficult to breath because of a problem in the chest or due to a blocked or runny nose? (Problem in chest only, a blocked or runny nose only, both or other; and (4) Did you seek advice or treatment for the illness from any source? (Yes, No or Don’t know). ‘In order to be eligible, the respondent had to answer “yes” to questions 1, 2 and 4, and “a problem in the chest” to question 3’.

A total of 173 children under 5 years old were screened, and 23 met the eligibility criteria. Out of these, 12 caregivers were purposively selected for in-depth interviews, out of which 11 participated in long-term participant observation. The selection criteria included having caregiving roles for children under five, living within the study area, and being willing to provide informed consent.

### Data collection

Data collection was conducted between April and September 2024. Participant observation for this research involved spending time with research participants in their homes, with each participant visited at least twice a month. The duration of each visit depends on the caregiver’s availability. Research assistants with experience in qualitative methods and a social science background were trained on how to note down observations and informal conversations.

In-depth interviews were conducted at the participants’ homes by the lead author using interview guides. Most interviews were conducted in Swahili. The interviews took approximately 30–50 min, all were audio recorded, except for one caregiver who opted out of recording; for this participant, longhand notes were taken instead. Participants were informed of the study objectives before the in-depth interviews and were thereafter requested to participate in participant observation, which included participation of the entire household. Throughout the follow-up period, several visits to the families were made during which informal interviews were conducted. During participant observation, an observation template was used to document daily childcare activities, healthcare-seeking practices and the environment in which these activities took place. A narrative guide facilitated informal interviews during conversations with caregivers.

The research team consisted of two research assistants, one with a Bachelor of Arts in Sociology and the other with a Bachelor of Arts in Anthropology. The lead researcher is a PhD candidate in Anthropology. The research team was trained in the research procedures to ensure consistency and rigour in data collection and analysis.

### Data management and analysis

Interviews were recorded and transcribed verbatim. Audio recordings in local languages were translated directly into English language during transcription. Quality checks involved comparing portions of the transcripts against the audio recordings. Comprehensive field notes were written after every participant observation. Field notes and transcripts were uploaded into Dedoose software for data management. Data analysis utilised a phenomenological hermeneutic approach, where transcripts were read multiple times, and significant statements were highlighted and coded. These codes were then grouped into themes that capture the essence of the participants’ experiences. The Hermeneutic Circle was employed, with the researcher engaging in a continuous process of interpreting the data, moving back and forth between the parts (individual statements) and the whole (overall understanding), to deepen the interpretation to get the ‘core essence’ of the lived experiences of the participants.

For our analysis, we focused on caregiver’s gender intersectionality in relation to experiences of care-seeking for childhood probable pneumonia. This included examining access to resources, gender roles and decision-making processes. In addition, we explored care-seeking dynamics that shape individual experiences.

We use illustrative quotes and provide textual descriptions of the qualitative data. Pseudonyms are used in textual descriptions and quotes.

### Trustworthiness

Credibility involved prolonged engagement with informants through participant observation. The multi-method approach facilitated data triangulation needed to validate information collected across the two different data sources. Conformability was achieved by reflecting upon interviews and narratives data obtained from participant observation and IDIs before analysis to minimise bias. Two researchers independently analysed the data and then discussed and reached an agreement by consensus. Finally, a homogeneous sample with a maximum diversity in demographic characteristics, marital status, education level, caregiver relationship with the child was ensured to maximise the findings’ transferability.

### Ethical considerations

Ethical clearance to conduct this study was obtained from the Maseno University Scientific and Ethics Review Committee (No. MSU/DRPI/MUSERC/01330/24), and National Commission of Science, Technology and Innovation (NACOSTI) (No. 765064). Written, informed consent was obtained from every participant enrolled in this study. Consent was obtained separately for participation in the in-depth interviews and participant observation. The information provided included in the consent forms include: the purpose of the study, the procedures to be followed; and the benefits or risks of this study. The information provided by the respondents was treated with much confidence and anonymity. Data are stored on a password-protected computer after identifiers have been removed.

## Results

### Participant characteristics

The study enrolled 12 caregivers, 11 females and 1 male, 1 female caregiver was a maternal grandmother to the child. Caregivers’ ages ranged from 22 years to 39 years with a median of 27.5 years. Refer to Table 1.

**TABLE 1:** Participants’ characteristics.

Pseudonym	Parents age (years)	Childs age (years)	Ethnicity	Educational level	Marital status	Occupation	Spouse occupation	Monthly income (Ksh)	No. of family members	No. of children	Children < 5 yrs
Atiela	26	2	Luo	O level	Single	Business	N/A	9000	5	2	1
Okumu	38	4	Luo	O level	Married	Business	Housewife	20 000	4	2	1
Pascalia	39	3	Luo	Primary	Single	Business	N/A	3000	3	2	1
Florence†	33	4	Luo	College	Separated	HRO	N/A	10 000	5	4	2
Mercy	24	4, 1	Luo	O level	Married	Online job	Teacher	8000	4	2	2
Siri	22	0.5 & 3, 5	Luo	Class 8	Married	Housewife	Business	Not known	5	3	3
Achieng	29	2	Luo	Bachelor’s Degree	Married	Teacher	Engineer	50 000	3	1	1
Atieno	34	4	Luo	Form 3	Married	Business	Driver	7000	4	2	1
Patricia	26	4, 5	Luo	O level	Married	Business	Business	20 000	4	2	2
Benta	24	2	Gisu	Class 6	Married	Business	Engineer	9000	3	1	1
Prisca	32	0.5‡ & 4‡	Luo	Bachelor’s Degree	Married	Teacher	HRO	50 000	6	4	4
Akumu	22	2	Luo	Form 3	Married	Business	County employee	5000	3	1	1

HRO, human resource officer; N/A, not available; No., number; Ksh, Kenyan Shilling.

† , Florence did not consent to participant observation;

‡ , Twins.

### Thematic findings

We present an intersectional gender perspective on lived experiences of caregivers of children with probable pneumonia.

Four overarching themes were identified: (1) Gendered roles and their impact on care-seeking experience; (2) gendered experience of the health system; (3) women’ access to resources and care-seeking experiences; and (4) decision making and care-seeking experiences.

### Gendered roles and their impact on care-seeking experience

Traditional as well as modifications or deviations from traditional gender roles (a heterogeneous mix) were evident in the caregivers’ narratives. In most cases, women typically managed all household chores in addition to engaging in income-generating activities. In contrast, most men worked in the informal sector with only a few in formal employment, working long hours outside the home, others working outside the locality, rarely assisting women in house chores and caregiving. Two women had formal employment and balanced between caregiving and work outside the home. Gender roles in this study were predominantly similar, whereas employed women hired women house-helps to assist with household chores and caregiving, those engaged in informal employment were involved in all household activities and caregiving while also involved in economic activities. Men provided financial and moral support although some women report that their husband did not support them emotionally and financially. Mothers were responsible for ensuring care and treatment for a sick child.

We observed that some men would avoid traditional roles such as caregiving, care-seeking and household chores which were perceived as ‘feminine’ tasks while others would defy societal expectation to take up more ‘feminine’ tasks. The broad range of responsibilities for women meant that some would decline hospitalisation to take care of their other roles at home.

Caregivers report that they rarely received support from their spouses and family members with chores, even when the children were sick. The women had to take care of children while also managing household responsibilities. They report that it is tiring and emotionally exhausting for them to care for a sick child while performing their other roles:

‘You just start from where you left. The place where you left that cup is where you will find it from.’ (Akumu, 22, 2, Luo, Form 3)

When one of Okumu’s children is unwell, he relies on his mother for cooking, as he identifies himself as a man; therefore, he doesn’t cook. His wife is responsible for care-seeking, while his mother provides food the family members admitted in the hospital and those at home:

‘When my child … if one of my children is unwell, you know definitely I am a man I can’t cook in my house and my mother is there. So I go and eat there and yet the mother [*wife*] goes with the child to the hospital. It is a support because I get food from the other side, the other child that is remaining also feeds from the other side [*mother’s house*]. When my wife … when the other child is admitted my mother cooks and takes food to the hospital. I think that is enough support.’ (Okumu, 38, 4, Luo, O level)

The multiple responsibilities of managing domestic tasks and care-seeking for children often place women in difficult situations, where they must make choices that are sometimes unfavourable to their children’s health. The societal expectation for women to fulfil multiple roles (caregiver, homemaker, and often, income-earner) can lead to significant stress and compromises in care decisions. These decisions are complex, as women must navigate the overwhelming challenge of balancing societal roles with the limited support available to them.

*Narrative 1*

Siri, a mother of three young children under 5 years, is responsible for most of the household chores. When her eldest son was sick, she declined admission because she needed to care for her younger child and manage [*the*] household responsibilities. Instead, she was given oral medications and allowed to go home. However, the child’s condition did not improve after which she had to take the child back to the hospital. This time she accepted admission because of the deteriorating condition of the child. After the child was given a bed and medication, she went back home to pick up her younger child. She had no one to either care for the younger child at home or provide care for the admitted child. She explains that she lost her samosa business because everything she had prepared to sell that evening was spoiled.

Her initial decision to decline admission was influenced by her caregiving responsibility and her desire to continue managing her small business. When the child’s condition deteriorated, Siri had to make a decision based on the severity of the child’s illness:

‘But the child … if he dies, he just goes like that. So, I had no choice but to get admitted. I left him when he had been put on drip. They told me to go back home and take things so that I go back with them to the hospital. I had to come back, take the younger one….’ (Siri, 22, 0.5 & 3, 5, Luo, Class 8)

Mercy’s case shows a shift from the traditional role observed from Siri’s narrative.

When Mercy’s older child was admitted with pneumonia, her husband took on the caregiver role during the entire hospital stay, while Mercy remained at home to care for their younger child. Mercy narrates: ‘Yes, I only went to hospital once when she was admitted. His brother was still very young’.

Men were largely absent from the day-to-day care of children, primarily fulfilling the role of financial providers for treatment and care-seeking. Some men worked away from home, leaving women to shoulder the full responsibility of caregiving on their own. In these circumstances, women are the sole care-seeking decision makers. The following story illustrates the gendered experience of a working mother with two sets of twins all under 5 years of age.

*Narrative 2*

Prisca is a mother of two sets of twins: the older set is 4 years old, and the younger set is 9 months old. She works as a teacher at a local school and has access to insurance. Her husband has a formal job that currently requires him to work away from home. Prisca lives in a rental house within a shared compound.

During our initial interview and subsequent visits, Prisca did not have a house girl to help with household chores and caring for her children. With her husband away, she managed all the responsibilities on her own. When one of the younger twins fell ill with a cough and running nose, Prisca went to the nearest pharmacy to buy medication, she considered the illness minor therefore not requiring a hospital visit. She explained that taking any of her children to the hospital was a challenge because she couldn’t leave any of them behind, and taking all of them together was impractical.

Recently, Prisca’s is getting support from a house help to assist her with household chores and care for the children. However, Prisca has fallen ill and she is now using crutches to move around. Despite her current condition, she is taking care of her children and also continues to go to work.

A few days prior to our visit, Prisca’s child fell ill at night, the child had very high fever but due to security reasons she waited until dawn to take the child to the hospital. Her house help took care of the rest of the children. Given her current condition, she traveled by motorbike, and the rider assisted her in alighting and carrying the child into the hospital. The child was diagnosed with an infection and treated with antibiotics.

In narrative 2, we observe that Prisca had to make several considerations before making care-seeking decision. She considered the severity of the illness and care for the other children. Despite her challenges, particularly with the demanding roles and poor health, she found ways to ensure that her children are taken care of while taking care of her other responsibilities.

### Gendered experience of the health system

Women generally seek care and provide care for young children during hospitalisation, although some men also took care of the children during hospitalisation. This meant that women were left to take care of the children during hospitalisation and deal with the challenges that arise within the healthcare system.

When Atieno’s child was admitted with severe pneumonia, she had an unpleasant experience. She struggled to communicate effectively with the nurses when she felt her son was deteriorating and needed immediate care. The nurses on duty kept sending her away when she wanted to raise her concern. When she couldn’t reach either of the nurses on duty, Atieno returned to her son’s bed and decided to wait until morning rounds to inform the doctor. Her husband, however, confronted the nurses, and this led to immediate action being taken.

Because of women’s primary care-seeking role, they had to deal with the day-to-day challenges of the health systems. These challenges included long waiting time, unfavourable conditions, poor communication, unfriendly staff and lack of drugs at the facility.

Siri describes the hassle of paying for services at the referral hospital, which employs a cashless payment policy that requires payment through mobile money transfer. She explains, ‘On reaching there, I did not have a phone and payment is usually made through a phone’. Because she did not have a phone, it was difficult for her to make the payment. Eventually, she was assisted in making the payment, but she had to pay 60 shillings, 10 shillings more than the required 50 shillings. Unfortunately, Akumu who did not have a phone either was not as lucky to get immediate assistance. She had to wait for approximately 6 h to pay for registration and to receive services at the hospital.

Caregivers also describe the challenging process of moving back and forth between departments at the hospital, each with its own distinct waiting times. They express frustration that after enduring these long waits (approximately 6 h), the hospital pharmacy often has no medications available. Most caregivers mentioned that this is a major deterrent to care-seeking at the hospital:

‘… they just give me Panadol; they tell me to go and look for Artemether and Lumefantrine (AL) [Antimalarial drug], if I go for AL maybe they have told me it is ksh150 or Ksh100 at that time I do not have that Ksh100 shillings, it is going to force me to return back to the house with the child and I have not treated him. That means I have not treated the child, right?’ (Akumu, 22, 2, Luo, Form 3)

A caregiver explains a negative experience she had at the hospital during her child’s hospitalisation. After her child was admitted with severe pneumonia for a month, they were unable to pay the hospital bill. During this time, they were moved out of the ward to make space for other sick children and were taken to a room full of beds without mattresses. She stayed there for a couple of days, as her husband looked for funds to settle the hospital bill.

### Women’s access to resources and care-seeking experiences

In the context of this study, families lived in an urban setting, most of them detached from the traditional *Luo* setup. In this environment, men maintained financial control over the household’s income and other resources. Apart from two women who had formal employment as teachers, the rest engaged in income-generating activities such as hawking, offering laundry and cleaning services, operating grocery stores, and informal work, the latter earning between Ksh100–350 per day. Women’s income was used to supplement their spouse’s, however, sometimes the money was not sufficient.

Many women, especially those with low income, mentioned their lack of access to financial resources to seek care at the hospital. When probed further, despite free healthcare initiative for children, respondents reported that they rarely received drugs at the hospital. Therefore, rather than waste time at the hospital, they preferred to buy drugs at the pharmacy with the little resources they had.

Accessing funds from the child’s father can be challenging for some women during a child’s illness. For example, Siri’s husband is the sole breadwinner since she had to close her business. She believes that although her husband may have money, he pretends not to. She says, ‘he usually has the money but he just pretends’. This portrayed a relationship dynamic, where all financial decisions are made by the husband who has the financial dominance in the relationship. In this case, relationship power dynamics because of male spouse financial dominance, restricts the women’s ability to make care-seeking decisions. Although she is able to recognise and determine the best course of action, she is sometimes unable to follow through on her decisions regarding healthcare because of financial constraint. It is only when the child’s condition worsened that her husband took action. ‘if he sees that the thing (the illness) is now serious is when he takes the step to buy medicine’.

As earlier stated in this article, women often supplement the household earnings by working outside the homes. In particular, some caregivers narrated how they work more than one job, for instance working as domestic workers and running a small business in the evening. Sometimes they are denied time off work to take their children to the hospital as was the case of Akumu, because they are afraid to lose their earnings they postpone taking the children to the hospital. Even women with formal employment are often afraid to request time off to take their sick children to the hospital, especially if the child has frequent bouts of illness. Therefore, they would prefer to use over-the-counter drugs.

*Narrative 3*

Akumu supplements the family income by selling fish for a fish vender in the evening and babysitting for the fish vender during the day. However, she earns only Ksh 100 per day, and sometimes this income goes unpaid by her employer due to poor business conditions. Akumu believes she needs to make money however little to supplement her husband’s income. She believes that she is now able to take her child to the hospital promptly when her husband cannot access money immediately. However, working long hours with little pay has come at a cost to her child’s health, as the child frequently falls ill due to Akumu’s inability to provide constant care due to her work obligations.

Akumu narrates in the following quote:

‘The little money that I have. Because obviously as a woman you should also have yours, so the little that I have is what I go to the hospital with. So, I just pay even if it is for the card, or maybe they wanted me to buy that syringe or something, I buy. If it gets to a point where I am stuck, I tell E’s* father here I am now stuck what do we do?’ (Akumu, 22, 2, Luo, Form 3)

Siri and Akumu share similar challenges regarding dependence on their husbands for financial support. They both believe it can be difficult for their husbands to act promptly when needed. Akumu laments that:

‘I must get angry, because I am hungry, the child is also hungry and medicine is also needed, you must just get angry. Because sometimes our men are also foolish, maybe he has money and he is waiting for you to call him is when he sends you the money. He will not send it to you at that time, he waits for you to look for him two or three times is when he sends you the money.’ (Akumu, 22, 2, Luo, Form 3)

Atieno, on the other hand, shares that whenever she can take care of her child’s financial needs, she just goes ahead and does so:

‘Okay, it depends. You just know how businesses are … sometimes business is bad you do not have money, sometimes you have money … something like that. So, when I have it, I will use it but if I do not … sometimes it finds my husband is far, he does not have money … if I have, I’ll just use it right.’ (Atieno, 34, 4, Luo, Form 3)

Atiela, a single mother of one, describes difficulty getting money from the father of her child. Often she makes the decision to use herbal medication because she cannot afford to buy conventional medicine.

### Decision making and care-seeking

In many households, mothers typically make decisions regarding seeking care for their children. This is because they have a deeper understanding of symptoms and can interpret them effectively because of spending more time with their children. However, care-seeking decisions are not made in isolation as they are interrelated with other socio-cultural, economic and structural factors, making them complex and multifaceted. Moreover, these decisions can change based on various extrinsic factors. The following example explains the intersection between gender dynamics and the care-seeking decision-making process.

Most women in this study made care-seeking decisions for their children. They thereafter inform their husbands about the child’s sickness and request for financial support to take the child to the hospital. The caregivers explain that they must inform their husbands of the decision to avoid conflicts within the households. However, if their husband delays in taking action, they take the initiative to bring the child to the hospital themselves:

‘It is me. Because I am the one that usually spends time with him, the father comes back at night, he will see him at night. So, I am the one to tell him that this child is sick, look at how you will take him to the hospital. And now when I tell him that and I see that he has delayed and the child is also suffering it will force me to rush.’ (Akumu, 22, 2, Luo, Form 3)

Atieno, like Akumu, makes care-seeking decisions for her young children based on the availability of money. If she has money, she will take her child to the hospital; if not, she will go to the local pharmacy. At the local pharmacy, they test for malaria, which she believed is more prevalent than other illnesses. For Atieno, ruling out malaria is the most important decision. She usually doesn’t consult her husband before making care-seeking decisions. Instead, she informs him of her decision, and he affirms it:

‘No, once I see that the child is sick, I take him or her to the hospital. If I do not have the money to take him to the hospital, I go to the local pharmacy. Because at the local pharmacy there is also a way that they test. If there is malaria they test, if it is not there, they tell you that maybe this is not malaria.’ (Atieno, 34, 4, Luo, Form 3)

Decision-making in care-seeking is influenced by various contextual factors beyond the individual characteristics of a caregiver. While socio-economic and cultural factors do play a significant role in care-seeking decisions, the specific roles and responsibilities of caregivers also heavily influence these decisions.

Caregiver’s role within their household and their work responsibilities impacted how and when they seek care for children. These roles often determine the amount of time and resources a caregiver can dedicate to care-seeking, which in turn affects their decisions. The following excerpt explains these dynamics:

‘Okay, I was having other activities so finding the time to go to the hospital was not easy. So, I just bought him … in fact when you left, in the evening when I got money, I bought him medicine. He has now been taking them because I have been busy for a while.’ (Atieno, 34, 4, Luo, Form 3)

Their busy schedule coupled with the time spent at the hospital and lack of medicine is a major deterrent to care-seeking at the hospital. They wonder why they should go through the challenges of seeking healthcare at the hospital when they still have to buy medicine at the local pharmacy.

As explained previously, Siri made the decision to take her child home against the doctor’s advice because she had a younger child to care for. When her child’s condition deteriorated, Siri decided to heed the doctor’s advice and have her child admitted to the hospital. Despite the lack of support from her husband, Siri made the decision to take care of both her children in the hospital.

Caregivers made decisions based on the severity of the illness; mild conditions such as colds and coughs are often managed at home or with over-the-counter medications, whereas more severe symptoms prompt a hospital visit. Even when financial resources are available, mothers may opt for convenience, such as purchasing medication from a pharmacy, if they are busy and want to avoid long waiting times at the hospital.

## Discussion

Our findings describe the lived experiences of women caregivers. The findings highlight the intricate interplay between gender roles, economic constraints and socio-cultural norms in shaping care-seeking experiences as has also been observed in other contexts.^19,20^

In this study, traditional gender roles were strongly evident, with women shouldering the primary responsibility for household chores, caregiving and care-seeking, even when engaged in competing income-generating activities. Previous reports from Africa and Asia underscore women work burden, particularly pronounced in rural areas, where women have triple responsibility for domestic, on-farm and off-farm work.^11^ Although our study was conducted in an urban setting, women’s responsibilities remained mostly the same. Instead of farming, the women engaged in economic activities outside the home. Our findings revealed that women frequently managed both domestic duties and economic activities, while men, although providing financial support were less involved in direct caregiving and household tasks. Similarly, previous studies indicated that mothers were the sole caregivers for young children, while fathers considered themselves as leaders of the family who took decision-making role, organising for logistics such as transport and arranging money for treatment.^30,31,32^ Our results are inconsistent with a study conducted in Cross River Nigeria, where fathers’ minimal involvement in child-rearing led to decreased financial support for the family.^33^ Therefore, women being the primary care-seekers meant that they had to balance between multiple gender roles ascribed to them.

Socio-cultural barriers and gender dynamics play an important role in care-seeking for children in our study. The study highlighted variations in how men adhered to or defied these traditional roles. Some men took up the role of caregiving at the hospital when a child was admitted, a role solely prescribed to women.^20^ This divergence illustrates the dynamic and heterogeneous nature of gender roles and the potential for shifts in caregiving responsibilities. The overarching pattern remained that women were the primary caregivers, a role that significantly influenced their experiences and challenges in seeking care for their children.^34^

Our study identified caregivers challenges related to navigating hospital structural environments and payment systems.^22^ Long waiting times, multiple service delivery points and a lack of medications resulted in negative experiences.^35^ Long waiting time led to the perception of poor quality of care. While these issues are not unique to women, more women than men seek care for young children. These challenges impact women’s economic activities, leading to a loss of income and opportunities to care for other children. As a result, these experiences and competing responsibilities often lead to delayed care-seeking or avoiding health facilities altogether. Beyond the healthcare costs associated with pneumonia,^36^ the impact of income loss because of missed work has not been sufficiently investigated.

Our study’s findings report that some women’s limited financial resources often meant relying on their husbands for money to seek medical care. This dependency delayed or hindered timely healthcare access, particularly when husbands were unable to provide financial support. However, barriers related to finances were never straightforward, often having many nuances. For instance, women only relied on money from the child’s father if they were unable to find resources for care-seeking on their own. While the lack of resources delayed or hindered care-seeking, it did not deter them from making healthcare decisions for their children. Therefore, we observe them making decisions, such as purchasing drugs from the local pharmacy, which is perceived as a cheaper alternative or making decision to take child to the hospital without male approval. These findings are consistent with previous studies,^20,32^ which show that women often accessed financial resources and made decisions regarding care-seeking. Urban dynamics contrast with those from rural areas where traditional gender roles and limited resources can restrict mothers’ ability to seek care independently, often leaving them reliant on family or community approval and support.^15,37^

Care-seeking decision-making process is complex. Past studies have documented various aspects of this process, including health beliefs, the involvement of the extended family, women’s lack of financial access and predominance of males in making care-seeking decisions among other factors.^11,28,37,38,39,40^ However, our study reveals that women who made final decisions for healthcare had to consider many factors beyond the availability of resources, their health beliefs, involvement of extended family closely related to their gender roles. Unlike other studies,^7,32^ the women in this study lived away from their relatives, leaving them solely responsible for their children’s care. Consequently, they would decline admission because they had to care for their other children at home or buy drugs from the pharmacy because they had no one to leave their other children with, or they were busy with income-generating activities, which led them to postpone care for their children. Our results indicate women’s healthcare decision-making is influenced by a complex interplay of factors, including gender roles and other nuance and subtle factors unique to each woman, which often leads them to prioritise other responsibilities over seeking timely healthcare for their children.

### Limitation

The key limitation of this study was the absence of men’s voices. During the ‘In depth Interviews’ only one male was available for the interview. During participant observation, we mostly found women during the visits. Whenever we found the men, they would excuse themselves to allow us to spend time with their wives. Because of these observations, the study plans to include the male voices by adding additional IDIs and conduct males-only and mixed-gender ‘Focus Group Discussions’.

## Conclusion

Our results explored the nuances of intersectional gendered experiences of care-seeking for children. Investigation into the ways in which women negotiate such complexities provides valuable insights into effective interventions. A multifaceted socio-structural framework approach ensures gender equality, economic empowerment and gender-sensitive healthcare practices in addressing these gender-related challenges. Recognising and responding to the unique experiences of women caregivers, policies and interventions are therefore in better position to support the vital contribution they make to their children’s health and well-being.

### Recommendations

Empowering women economically: There is a need for policies that enhance financial independence for women, including microfinance and economic empowerment to enable them to make independent decisions regarding their health.

Promote gender-sensitive healthcare: Health systems should apply gender-sensitive responses to the special challenges that confront women caregivers. Training healthcare providers on respectful and equitable engagement with women can enable communication and health outcomes.

Encourage men to be more involved in caregiving: A shared caregiving responsibilities programme would lighten the burden that falls squarely on female caregivers’ shoulders and help challenge traditional gender roles in caregiving. Community-based interventions that involve men in caregiving activities have the potential to stimulate supportive and equitable household dynamics.

Improve access to and quality of health services: Ensuring appropriate health infrastructure development and the availability of essential drugs and services at public health facilities could result in reduced dependence on out-of-pocket expenses, with improved perceptions related to quality and timely access to medical care for children.
